# Lethality risk markers by sex and age-group for COVID-19 in Mexico: a cross-sectional study based on machine learning approach

**DOI:** 10.1186/s12879-022-07951-w

**Published:** 2023-01-11

**Authors:** Mariano Rojas-García, Blanca Vázquez, Kirvis Torres-Poveda, Vicente Madrid-Marina

**Affiliations:** 1 Center for Research on Infectious Diseases, Instituto Nacional de Salud Pública, Cuernavaca, 62100 Mexico; 2 Instituto de Investigaciones en Matemáticas Aplicadas y en Sistemas, Universidad Nacional Autónoma de México, Mexico City, 04510 Mexico; 3 CONACyT-Instituto Nacional de Salud Pública, Av. Universidad 655, Santa María Ahuacatitlán, 62100 Cuernavaca, Mexico

**Keywords:** COVID-19, Lethality risk markers, Machine learning, Mexico

## Abstract

**Background:**

Mexico ranks fifth worldwide in the number of deaths due to COVID-19. Identifying risk markers through easily accessible clinical data could help in the initial triage of COVID-19 patients and anticipate a fatal outcome, especially in the most socioeconomically disadvantaged regions. This study aims to identify markers that increase lethality risk in patients diagnosed with COVID-19, based on machine learning (ML) methods. Markers were differentiated by sex and age-group.

**Methods:**

A total of 11,564 cases of COVID-19 in Mexico were extracted from the Epidemiological Surveillance System for Viral Respiratory Disease. Four ML classification methods were trained to predict lethality, and an interpretability approach was used to identify those markers.

**Results:**

Models based on Extreme Gradient Boosting (XGBoost) yielded the best performance in a test set. This model achieved a sensitivity of 0.91, a specificity of 0.69, a positive predictive value of 0.344, and a negative predictive value of 0.965. For female patients, the leading markers are diabetes and arthralgia. For males, the main markers are chronic kidney disease (CKD) and chest pain. Dyspnea, hypertension, and polypnea increased the risk of death in both sexes.

**Conclusions:**

ML-based models using an interpretability approach successfully identified risk markers for lethality by sex and age. Our results indicate that age is the strongest demographic factor for a fatal outcome, while all other markers were consistent with previous clinical trials conducted in a Mexican population. The markers identified here could be used as an initial triage, especially in geographic areas with limited resources.

**Supplementary Information:**

The online version contains supplementary material available at 10.1186/s12879-022-07951-w.

## Background

Among middle-income countries, Mexico is one of the most severely affected by COVID-19. According to recent estimates, the burden of disease due to COVID-19 in Mexico amounts to 2,165,424.5 disability-adjusted life years (DALYs) [1]. Mexico has the fifth highest number of COVID-19 deaths in the world, after the United States, Brazil, India and Russia [2]. In October 2022, nearly 6.5 million deaths from COVID-19 were reported worldwide and 330,227 were reported in Mexico, with a cumulative weekly mortality rate of 253 per 100,000 population [3]. Great heterogeneity has been reported among age- and sex-adjusted case fatality rates (CFR) in Mexican adults with a positive diagnosis of SARS-Cov-2, ranging from 4.6% in private hospitals to 18.9% in public facilities [4]. These divergences in mortality rates are largely due to inequalities in the Mexican population [5] and the response to the pandemic by Mexican health authorities [6]. Therefore, markers of risk of death are urgently needed to allow clinicians to perform initial triage in patients with COVID-19 and to anticipate a fatal outcome, especially in the most socioeconomically disadvantaged regions of Mexico.

Several studies around the world have evaluated factors associated with severe disease and death from COVID-19 [7, 8]. Also, predictive models for COVID-19 using laboratory tests or imaging markers have been proposed [9–11]. However, their potential use by clinicians, especially in low-resource healthcare settings, is limited [12]. Thus, a model to predict the risk of death using data that are readily available in clinical areas, such as patient signs and symptoms, would be extremely valuable in those areas of Mexico where access to specific tests is limited.

This study is aimed to assess risk markers, stratified by sex and age-group, for COVID-19 lethality in Mexico by a machine learning (ML) approach. Particularly, we evaluated various supervised classification models to predict lethality in patients diagnosed with COVID-19 using clinical data. Then, we applied an interpretability approach to identify those markers that increase lethality risk, by sex and age-group.

## Methods

### Study design and data source

This is a cross-sectional study using data collected from an open-access database. This database includes symptoms and signs of patients reported to the Epidemiological Surveillance System for Viral Respiratory Disease (SISVER).

SISVER collects data on patients with suspected COVID-19. The cases are reported using the questionnaire “Epidemiologic study of suspected viral respiratory disease”, available on the official site of the Federal Health Secretariat in Mexico.^1^ Typically, the first-contact clinician completes the form with patient demographics, comorbidities, symptoms, signs of illness, and medication prior to hospital admission. If the patient is admitted to an intensive care unit (ICU), the doctor adds this admission to the questionnaire. The patient’s outcome, either death or discharge, is also recorded. Unfortunately, the evolution of symptoms and signs is not recorded.

SISVER data are processed and published in an open-database available on http://covid-19.iimas.unam.mx/. In this database, data on symptoms, signs, comorbidities, and prior medication were recorded as categorical variables. These variables were recorded with three values, “Yes,” “No,” and “Not known.” For instance, if a patient reported chronic obstructive pulmonary disease (COPD), then the value for this comorbidity was recorded as “Yes;” if the patient stated they do not have COPD, the value was recorded as “No;” otherwise, it was coded as “Not known.” All data were collected at hospital admission. User registration providing name, institution, position, and email is required to access data.

### Patient selection

The database included information on 5,490,290 suspected COVID-19 cases that were admitted to the hospital from June 9, 2020, to March 1, 2021. Inclusion criteria were, patients with COVID-19 confirmed by RT-PCR living in the State of Morelos who were not undergo intubated and were not admitted to the ICU. A total of 11,564 patients were selected. Baseline characteristics of patients are described in Table 1. Continuous variables are reported as mean and standard deviation, and categorical variables are reported as percentages. From all included patients, 46% were females and 54% were males, with an average age of 49.12 ± 17.53 years. The most common symptoms in both sexes were cough, fever, headache, and myalgia. Lethality rates were 5% for females and 9% for makes. All data used herein are publicly downloadable from this open-access database.

**Table 1:** Characteristics of patients

Clinical variable	Males	Females	Total	*P*-value*
Demographic				
Sex	6245 (54%)	5319 (46%)	11,564 (100%)	< 0.001
Age	50.61 ± 17.68	47.37 ± 17.19	49.12 ± 17.53	–
Age-group				
< 19	134 (1%)	140 (1%)	274 (2%)	–
20—29	657 (6%)	696 (6%)	1353 (12%)	–
30—39	1091 (9%)	1127 (10%)	2218 (19%)	–
40—49	1149 (10%)	1072 (9%)	2221 (19%)	–
50—59	1150 (10%)	951 (8%)	2101 (18%)	–
60—69	1034 (9%)	700 (6%)	1734 (15%)	–
> 70	1030 (9%)	633 (6%)	1663 (14%)	–
Comorbidities				
Hypertension	1567 (14%)	1225 (11%)	2792 (24%)	< 0.001
Diabetes	1266 (11%)	997 (9%)	2263 (20%)	< 0.001
Obesity	1005 (9%)	954 (8%)	1959 (17%)	0.487
Smoking	547 (5%)	190 (2%)	737 (6%)	0.004
CKD	219 (2%)	126 (1%)	345 (3%)	< 0.001
Cardiovascular diseases	150 (1%)	106 (1%)	256 (2%)	< 0.001
COPD	101 (1%)	129 (1%)	230 (2%)	< 0.001
Asthma	88 (1%)	132 (1%)	220 (2%)	0.042
Immunosuppression	36 (0%)	38 (0%)	74 (1%)	0.016
Symptoms				
Cough	5469 (47%)	4553 (39%)	10,022 (87%)	< 0.001
Headache	5211 (45%)	4609 (40%)	9820 (85%)	< 0.001
Fever	5429 (47%)	4353 (38%)	9782 (85%)	< 0.001
Myalgia	4677 (40%)	3979 (34%)	8656 (75%)	0.007
Arthralgia	4444 (38%)	3719 (32%)	8163 (71%)	< 0.001
Odynophagia	3771 (33%)	3281 (28%)	7052 (61%)	0.019
General discomfort	3707 (32%)	2877 (25%)	6584 (57%)	< 0.001
Chills	3297 (29%)	2760 (24%)	6057 (52%)	0.310
Dyspnea	3113 (27%)	2025 (18%)	5138 (44%)	< 0.001
Sudden onset of symptoms	2191 (19%)	1802 (16%)	3993 (35%)	0.019
Chest pain	2079 (18%)	1732 (15%)	3811 (33%)	< 0.001
Rhinorrhea	1796 (16%)	1760 (15%)	3556 (31%)	< 0.001
Diarrhea	1301 (11%)	1155 (10%)	2456 (21%)	< 0.001
Anosmia	933 (8%)	995 (9%)	1928 (17%)	< 0.001
Irritability	940 (8%)	816 (7%)	1756 (15%)	0.060
Dysgeusia	807 (7%)	915 (8%)	1722 (15%)	< 0.001
Polypnea	1065 (9%)	650 (6%)	1715 (15%)	< 0.001
Abdominal pain	741 (6%)	756 (7%)	1497 (13%)	0.551
Conjunctivitis	638 (6%)	580 (5%)	1218 (11%)	< 0.001
Vomiting	424 (4%)	459 (4%)	883 (8%)	0.240
Cyanosis	239 (2%)	137 (1%)	376 (3%)	< 0.001
Medication				
Use of antipyretics	3268 (28%)	2793 (24%)	6061 (52%)	0.014
Days elapsed from the onset of symptoms to the start of medical care	3.51 ± 2.42	3.42 ± 2.37	3.47 ± 2.40	-
Lethality (death/no death)	1084 (9%)	538 (5%)	1622 (14%)	-

*COPD* chronic obstructive pulmonary disease, *CKD* chronic kidney disease. Immunosuppression: due to diseases like cancer, immunosuppressive drugs, and transplantation

*P-value on chi-square test

**Fig. 1:**
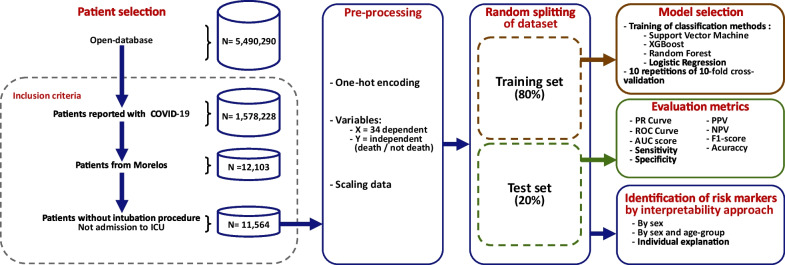
Process to identify lethality risk markers for COVID-19 patients

The process of training and evaluation of ML methods to identify markers of lethality risk in the population under study is shown in Fig. 1. It included patient selection, preprocessing, model selection, final performance evaluation, and identification of risk markers for lethality.

### Preprocessing

To select the clinical variables for the dataset, those fields in the database with more than 90% of missing data were excluded. Thus, 34 variables with no missing values were included. One-hot encoding was used for all categorial variables. The time of elapsed from the onset of symptoms to the start of medical care was estimated in days. Age and time elapsed were regarded as continuous variables. Mortality, selected as the independent variable, was used to predict lethality (survival or death). All other variables were regarded as dependent. A brief description of all clinical variables used is shown in Additional file 1. Data were transformed as a standard Z distribution. The models were trained with 80% of data, and the remaining 20% was used for testing.

### Model selection

Four machine learning classification methods were used in this study: Logistic regression (LR), support vector machine (SVM), random forest (RF), and eXtreme gradient boosting (XGBoost). For hyperparameter selection, we relied on a grid search based on 10 repetitions of stratified tenfold cross-validation on the training set.

The hyperparameters evaluated for each method were set as follows. For LR, saga and liblinear optimizer were used [13]. For weight penalization, ℓ1, ℓ2, and elastic net norms were considered [14]. Ratios of 0.1, 0.2, 0.4, 0.5, 0.6, 0.7, 0.8, and 0.9 were used for ℓ1. Different values for the strength C of penalization were considered on a logarithmic scale, including − 3, 3, and 7. For SVM, a penalization value ranging from − 4 to 4 was considered [14]. For RF, the maximum number of features was set using a base-2 logarithm for each split, and impurity decrease was set to 1e − 4 [15]. For XGBoost, dropout rates of 0.03 and 0.5 were evaluated, and the learning rate was considered as 0.03, 0.05, and 0.1 [16]. For both RF and XGBoost, 200, 250, and 300 estimator trees were used, with a maximum depth of 5 and 6 nodes. All these hyperparameters were evaluated as usually during the training of ML methods.

The selection criterion for training was the area under the receiver operating characteristic curve (ROC AUC). The model with the highest mean of cross-validated AUC was selected. All metrics computed to measure model performance on the test dataset are shown in Table 2.

**Table 2:** Metrics evaluated on test set

Metrics	Formula	Description
Sensitivity (recall)	$\frac{TP}{ TP + FN}$	Measures the proportion of diseased patients with a positive test result
Specificity	$\frac{TN}{ TN + FP}$	Measures the proportion of patients without disease with a negative test result
Positive Predictive Value (PPV)	$\frac{TP}{TP + FP}$	Measures the ability of the classifier not to label as positive a sample that is negative
Negative Predictive Value (NPV)	$\frac{TN}{TN + FN}$	Measures the ability of the classifier not to label as negative a sample that is positive
F1-score	$2 *\frac{Recall * Precision}{Recall + Precision}$	It is the harmonic mean of Precision and Recall
Accuracy	$\frac{TP+ TN}{TP+FP+FN+TN}$	Is the percentage of patients classified correctly with respect to the total of cases examined

*TP* true positive, *FN* false negative, *TP* true negative, *FP* false positive

As our dataset is imbalanced, the ROC curve and the Precision–Recall curve (PR) were computed on the test. The ROC curve analyzes the sensitivity and specificity achieved by the model. Meanwhile, the PR curve shows performance based on false-positive and false-negative rates.

### Identification of risk markers by the interpretability approach

An interpretability approach was used to identify risk markers. In particular, the Shapley Additive explanations (SHAP) algorithm [17] was used to interpret the output of predictive models. According to Molnar [18], SHAP explains the prediction of an instance $x$ by computing the contribution of each feature to the prediction. The SHAP algorithm computes Shapley values from coalitional game theory, where the game is the prediction task for a single instance, and the players are the values of the features. This algorithm computes the contribution of each player to the game. The Shapley value $\phi j(val)$, the payout that a player $j$ receives for the game, is computed as follows:

$${\phi }_{j}(val)={\sum }_{S\subseteq \{1,...p\}\backslash \{j\}}\frac{|S|!(p-|S|-1)!)}{p!} (val (S\cup \{j\}-val(S))$$

where $S$ is a subset of the features used in the model, $val$ is the vector of feature values of the instance to be explained, and $p$ is the number of features. The Shapley value is the average marginal contribution of a feature value across all possible coalitions. The SHAP algorithm has already been used to calculate markers for diagnosis [19] and mortality by COVID-19 [20], for hypoxemia prevention [17], and for mortality after an infarction [21]. All algorithms were written in Python v.3.8. Scikit-learn,^2^ pandas,^3^ numpy,^4^ and Jupyter Notebook^5^ libraries were also used. Source code is available at https://github.com/rojas-mariano-salvador/Lethality-markers-for-COVID-19.

## Results

### Performance of lethality prediction models

**Fig. 2:**
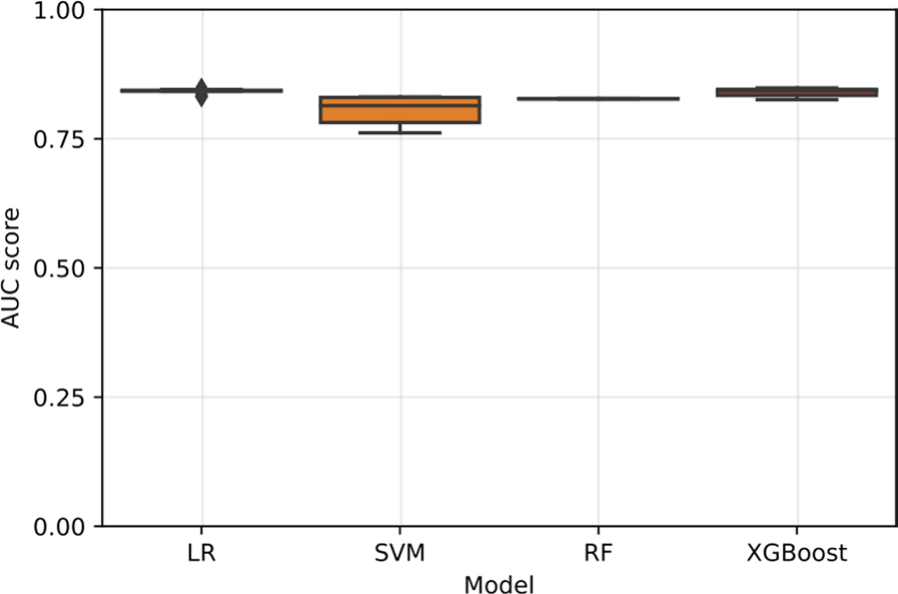
Comparison of machine learning classification models using 10 repetitions of stratified tenfold cross-validation

The dispersion of AUC scores in each cross-validation fold for each model evaluated on the validated set are shown in Fig. 2 and Additional file 2. As shown, XGBoost performed better in terms of discrimination than the rest of the models during training. XGBoost achieved the highest performance, outperforming LR, SVM, and RF. The best parameters found for XGBoost were as follows: maximum depth = 5, estimator = 200, dropout rate = 0.3, and learning rate = 0.03.

**Fig. 3:**
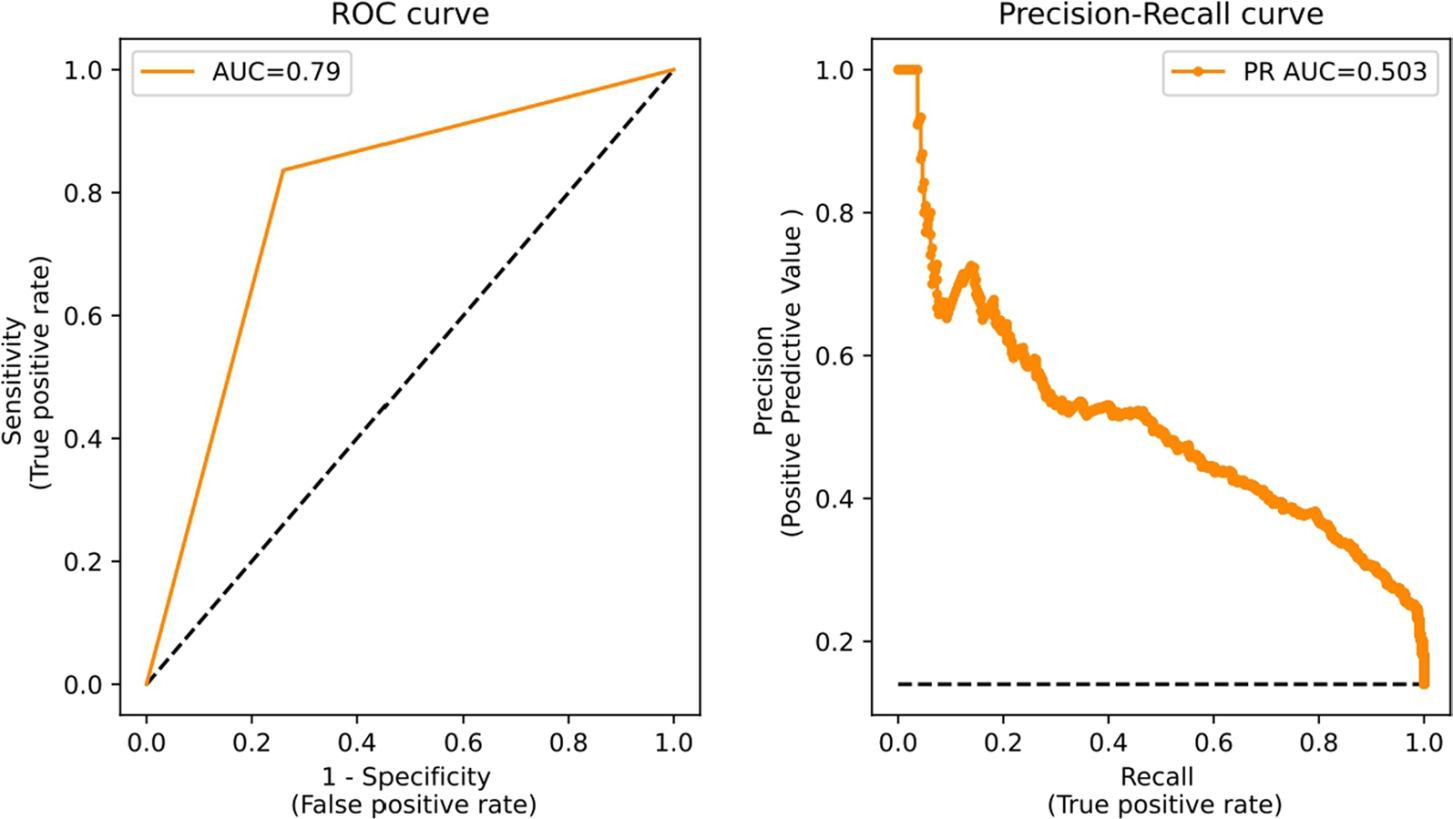
Curves for XGBoost model on test set. ROC curve (left) and PR curve (right)

Finally, the metrics shown in Table 3 were computed, and ROC and PR curves for the selected XGBoost model on the test set were plotted (Fig. 3). Overall, the model achieved an ability discriminative AUC score of 0.79 and a precision-recall of 0.503; sensitivity was 0.836 and specificity was 0.74.

**Table 3:** Performance of XGBoost model on test set

Metrics	Performance
Positive predictive value	0.344
Negative predictive value	0.965
Sensitivity	0.836
Specificity	0.74
F1-score	0.487
Accuracy	0.754

### Lethality risk markers by sex and age-group

**Fig. 4:**
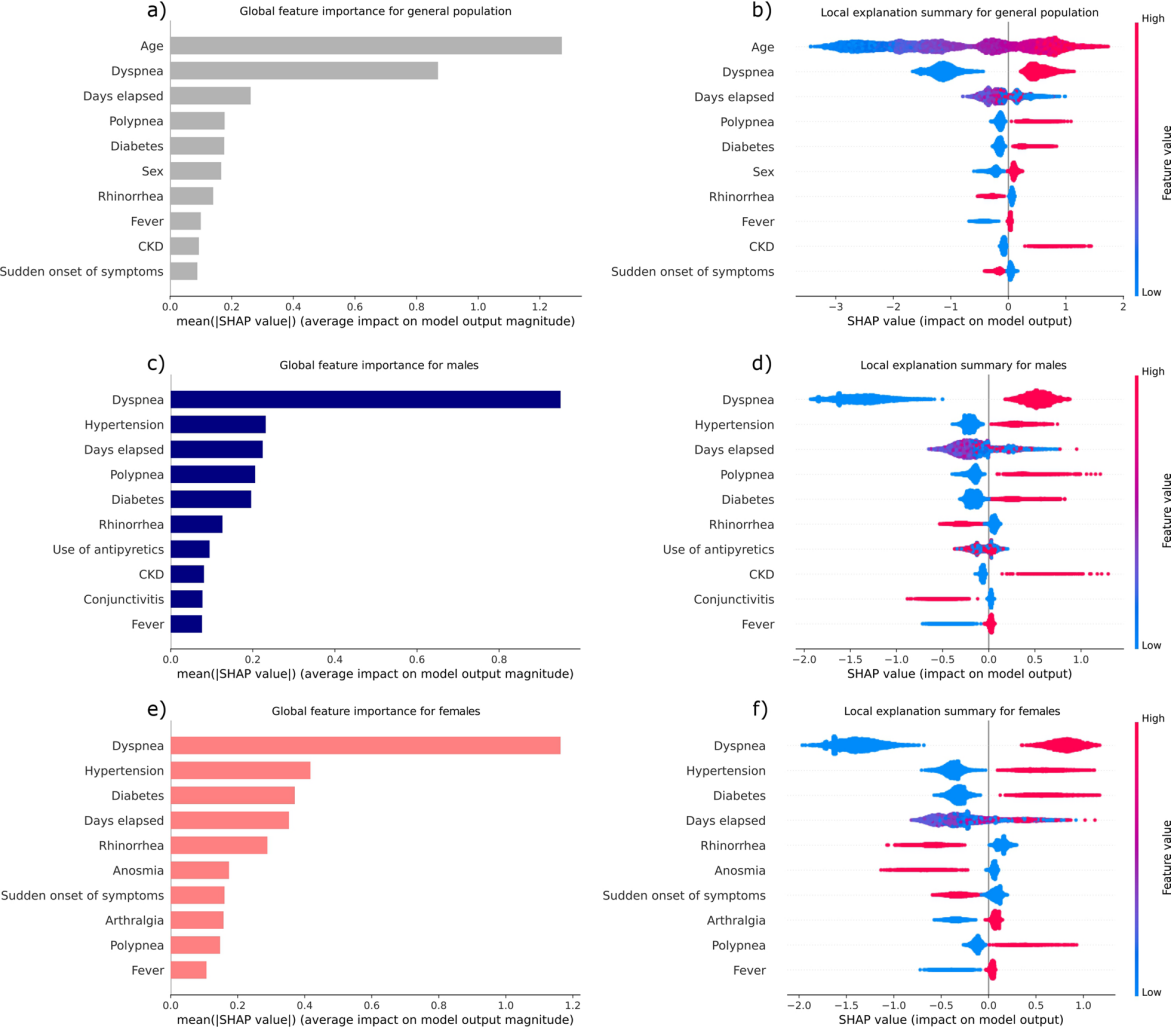
Risk markers in the general population and in male and female patients

The markers found to increase the lethality risk by sex through an interpretability approach are shown in Fig. 4. Bar plots (left) show the features in order of importance. On the other hand, beeswarm plots (right) show the impact of each feature value on the model output. The SHAP value is shown in the *x*-axis: larger positive SHAP values increase lethality risk, whilst larger negative SHAP values decrease the risk. Each patient is reported as a dot in this plot. Multiple dots in the *x*-axis shape a density. Colors indicate the value of each feature. Larger values are plotted in red, whilst smaller values are plotted in blue.

First, risk markers were evaluated in both sexes. Age was the main marker that increased the risk of lethality. As shown in Fig. 4b, lethality increased with age (larger positive SHAP values on the *x*-axis). The presence of dyspnea, polypnea, and diabetes also increased the risk.

Then, risk markers were analyzed by sex. Chronic kidney disease (CKD) (Fig. 4c and d) was a good marker for male patients, and arthralgia was good for females (Fig. 4e and f). Common markers for both sexes were dyspnea, hypertension, polypnea, diabetes, and fever.

The markers with the greatest contribution to lethality were dyspnea and hypertension for males and females; however, the third most relevant marker for males was polypnea, while diabetes was the third for females. In both groups, fever was the marker with the lowest contribution to lethality.

Additionally, as shown in Fig. 4d and f, rhinorrhea in males and females, conjunctivitis in males, as well as a sudden onset of the disease and anosmia in females decreased the risk on lethality.

A summary of lethality risk markers by sex and age-group is shown in Table 4. Notably, respiratory compromise signs such as dyspnea and polypnea were the most frequent risk markers in all age groups.

**Table 4:** Risk markers by sex and age-group

Patients stratified by age	Risk markers for males	Risk markers for females	In common
20–29*	Dyspnea, polypnea, days elapsed, chest pain, chills, hypertension, Smoking	–	–
30–39	Chest pain, CKD, obesity	Diabetes, odynophagia, diarrhea, chills	Dyspnea, polypnea, hypertension
40–49	Chills	Hypertension, vomiting	Dyspnea, days elapsed, polypnea, diabetes, CKD
50–59	Chest pain, odynophagia, chills	Diabetes, general discomfort, anosmia	Dyspnea, polypnea, use of antipyretics
60–69	Chest pain, myalgia, CKD, use of antipyretics	Diabetes, cough, arthralgia	Dyspnea, hypertension, vomiting
≥ 70	Chest pain, myalgia, smoking	Hypertension, chills, CKD, arthralgia	Dyspnea, polypnea, use of antipyretics

*COPD* chronic obstructive pulmonary disease, *CKD* chronic kidney disease. Days elapsed: elapsed days from symptom onset until medical care. * The age group 20–29 years was discarded in the analysis because only one death was observed in this group and the model could not be trained to identify markers for comparison with males

Chest pain was a risk marker for male patients in various age groups. On the other hand, diabetes was the most frequent risk marker for females in various age groups; arthralgia was found to be a marker in females aged 60 years or older.

As shown in the swarm graphs in Additional file 3, the presence of some signs of the disease actually decreases the risk of lethality. Such is the case of odynophagia in male patients younger than 50–59 years old, or rhinorrhea and sudden disease onset in females in some age groups. In addition, the use of antipyretics increased the risk in males and females aged 50 years or older. Unexpectedly, we found that those patients who seek care on time increase the risk on lethality.

The distribution of deaths by sex and age-group is shown in Table 5. As shown, deaths were more frequent in older patients, with 96% of deaths in males and 95% in females in age groups ≥ 40 years. Interestingly, the model assigned a person an initial probability of death as a function of his or her age group, and this initial probability increases with age.

**Table 5:** Distribution of deaths by sex and age-group

Patients stratified by age	Males		Females	
	Death	Survival	Death	Survival
≤ 19	2 (0%)	132 (3%)	3 (1%)	137 (3%)
20–29	14 (1%)	643 (12%)	0 (0%)	696 (15%)
30–39	38 (4%)	1053 (20%)	20 (4%)	1107 (23%)
40–49	88 (8%)	1061 (21%)	30 (6%)	1042 (22%)
50–59	203 (19%)	947 (18%)	104 (19%)	847 (18%)
60–69	310 (29%)	724 (14%)	164 (30%)	536 (11%)
≥ 70	429 (40%)	601 (12%)	217 (40%)	416 (9%)
Total	1084	5161	538	4781

The contribution of clinical history to prediction was also analyzed. The SHAP algorithm was used to analyze data of six patients in the group with significant lethality (50–70 years).

**Fig. 5:**
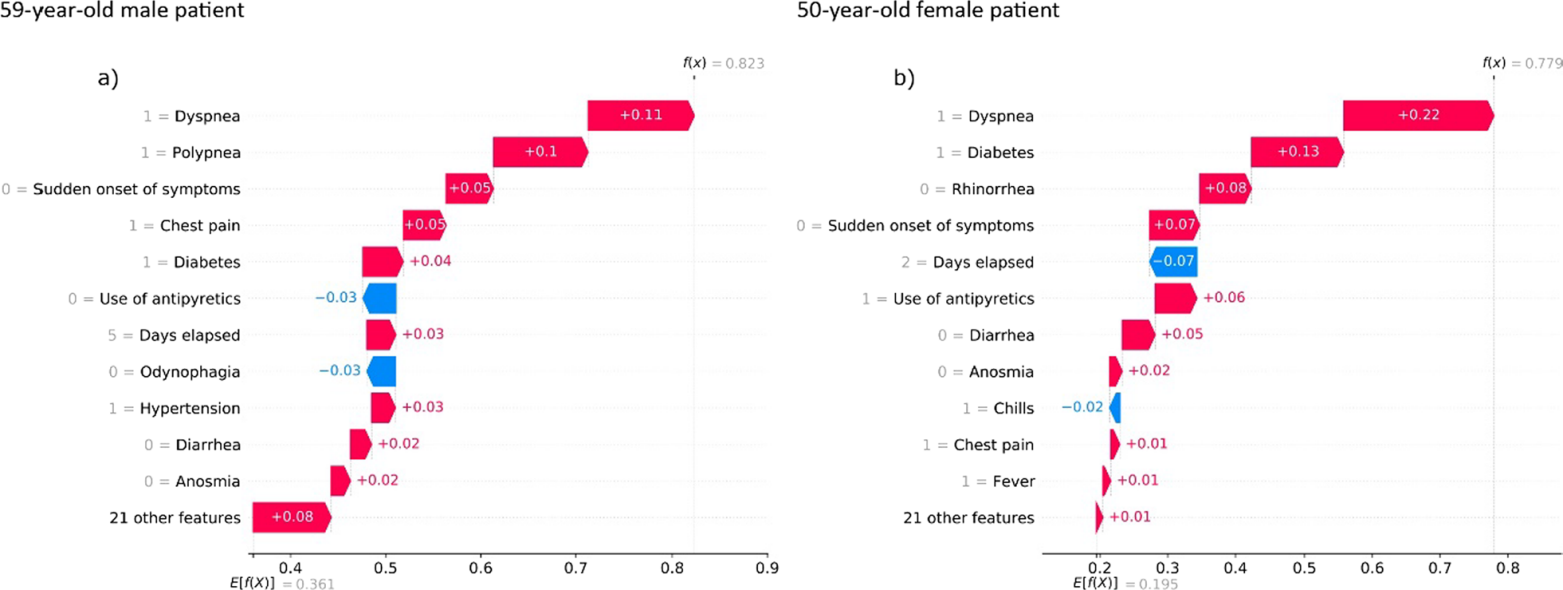
Individual prediction for a male and a female patient in the 50–59 age group, who died from COVID-19

**Fig. 6:**
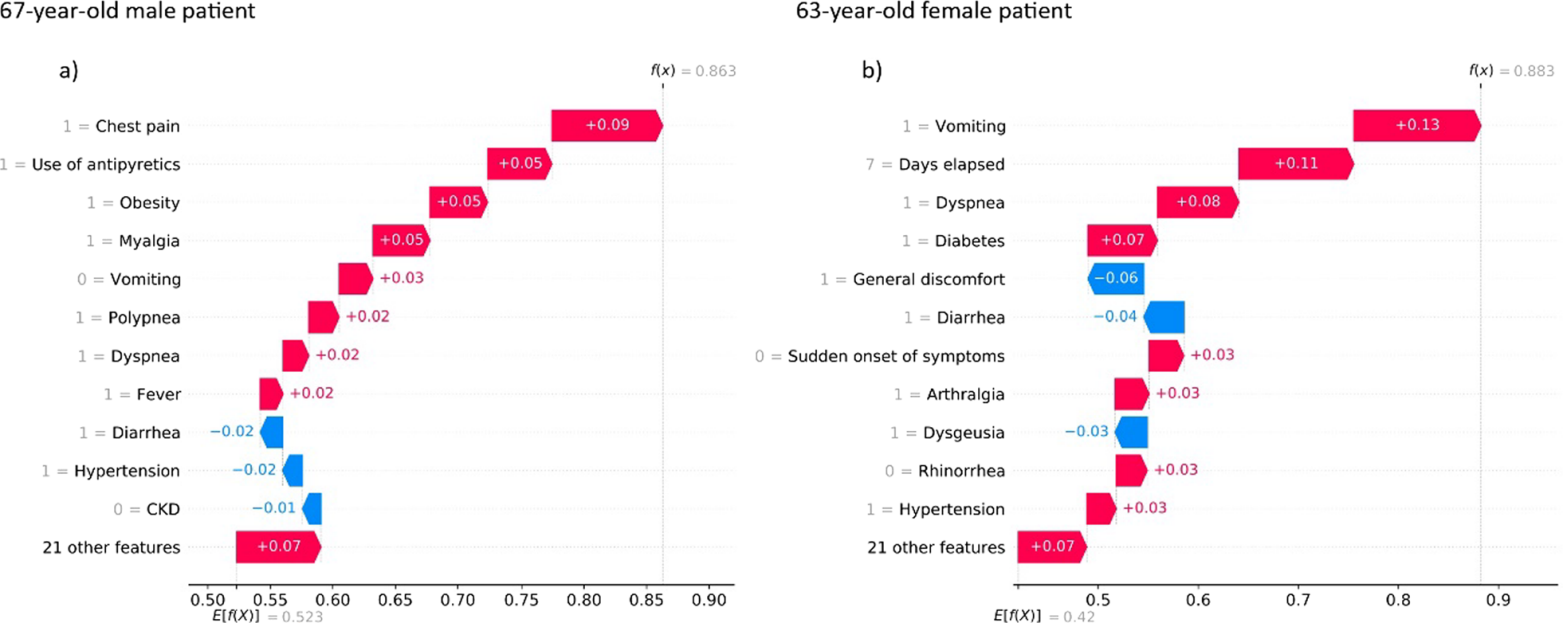
Individual prediction for a male and a female in the 60–69 age group who died from COVID-19

**Fig. 7:**
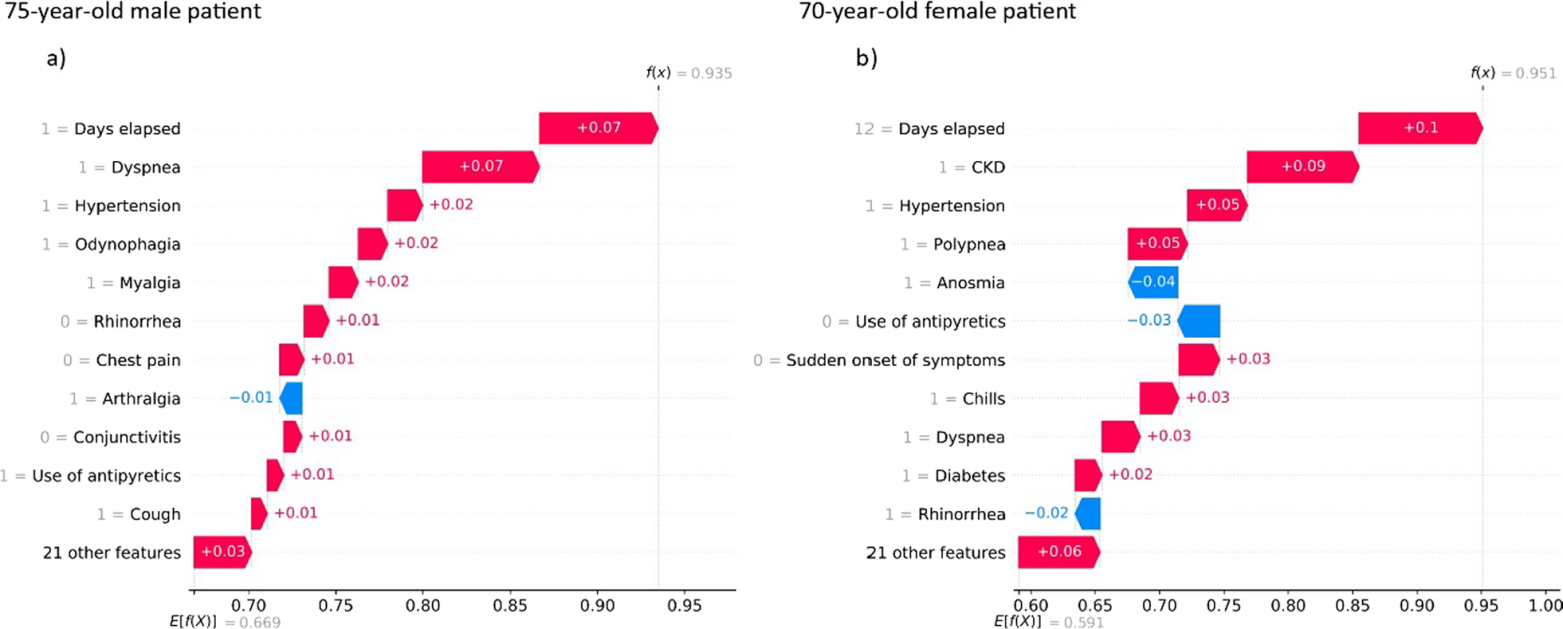
Individual prediction for a male and a female in the age group ≥ 70 years who died from COVID-19

Figures 5, 6, 7 show individual predictions for three patients who died from COVID-19. In particular, these plots show the probability of death or lethality risk computed by the model and denoted as $f(x)$. The average output probability from all the patients is denoted as $E[f(x)]$, which represents the baseline risk according to age-group and sex. The clinical variables that increased the risk of lethality ranked in descending order. The contribution of each variable to the prediction is also shown as positive (red) or negative (blue).

The clinical data of a 59-year-old male patient who died from COVID-19 are shown in Fig. 5a. In this case, the risk of lethality was 0.823. The markers that increased the risk were dyspnea, polypnea, chest pain, and diabetes. In contrast, the clinical data of a 50-year-old female patient who also died are shown in Fig. 5b. The risk in this case was 0.779, and the markers that increased the risk were dyspnea, diabetes, and the use of antipyretics.

Similarly, the case of a 67-year-old male patient is shown in the left panel (a) of Fig. 6, and the case of a 63-year-old female patient is shown in the right panel (b). According to our model, the male had a risk of death of 0.863; the risk markers that led to this result were the presence of chest pain, obesity, myalgia, and the use of antipyretics, and to a lesser extent, polypnea, dyspnea, and fever. The risk of death for the female patient was 0.883; the markers that increased the risk were vomiting, dyspnea, diabetes, and a period of 7 days elapsed from the onset of the disease until medical care was given; arthralgia and hypertension also contributed of lethality.

The cases of a 75-year-old male (left panel) and a 70-year-old female (right panel) are shown in Fig. 7. The male had a risk of death of 0.935; because the manifestation of the dyspnea, odynophagia and myalgia, to have hypertension and a time of one days elapsed from the onset of the disease to medical care. The female had a risk of death of 0.951, with a time of 12 days elapsed from the onset of the disease to medical care, to have CKD, hypertension and diabetes as well as manifestation of the polypnea being the major contributors to a fatal outcome.

In all cases, the ground truth of all these patients was that they died. The interest to analysis these cases is to validate the performance of XGBoost model for distinguishing lethality and identifying the risk markers that increase the risk.

Overall, we observed that the variables that contributed to lethality are the symptoms related to respiratory involvement (dyspnea and polypnea). Also, hypertension was identified as a comorbidity that contributes to lethality.

In particular, chest pain was a marker that contributed to lethality in males. The use of antipyretics was also recurrent as a marker in males, in contrast with females.

On the other hand, even though diabetes was identified as a risk marker in both sexes, disaggregated data showed a higher frequency and weight in lethality for females.

## Discussion

### Summary

Since case lethality estimates the frequency of death among confirmed cases and it depends on factors such as the sensitivity of the case detection system, the responsiveness of health services, and a timely treatment [22], an early detection of risk markers for COVID-19 case fatality could favor a better outcome for infected patients.

The use of machine learning models to predict COVID-19 lethality in a stratified Mexican population has been little explored. This work is aimed to build models based on demographic and clinical variables (symptoms, signs, and comorbidities). These data are often available for practitioners in settings where resources, technology, and access to specialized personnel are limited, a situation common in a middle-income country like Mexico. The results of this study provide valuable information on the relationship between risk markers stratified by sex and age-group and COVID-19 lethality in Mexico.

### Comparative with the state of the art

Efforts have been made to develop and validate models for predicting death in Mexican patients with COVID-19 using demographic and patient history factors [23, 24]. The PH-Covid19 scoring system was developed by Mancilla-Galindo et al. using data sets from the Mexico COVID-19 Epidemiological Surveillance Study and validated in cohorts including outpatients and inpatients. The multivariable Cox regression model includes eight predictors and yielded a discrimination of death of 0.8 evaluated by Harrell’s C-statistic [23]. Our study was stratified by sex and age-group, and it is noteworthy that our variables were not limited to comorbidities, sex and age, but we also included signs and symptoms.

Several diagnostic and prognostic models have been proposed for COVID-19, for instance the studies carried out by Aktar et al. [25] and Cini Oliveira et al. [26]. Both approaches evaluated several machine learning models to identify the best predictors of COVID-19 mortality based on demographic data, symptoms and signs, and comorbidities. Like our proposal, both studies use the SHAP approach to distinguish the markers that increase the risk of mortality. On the one hand, Aktar et al., evaluated the algorithms of RF, SVM, XGBoost, Decision Tree, Gradient Boosting Machine, and Light Gradient Boosting Machine (LightGBM) for predicting of mortality. The LightGBM model achieved a discrimination of 0.89, evaluated by the AUC score. On the other hand, Cini Oliveira et al. evaluated the algorithm of XGBoost for predicting of mortality achieved an AUC = 0.94. While our model achieved a lower discrimination power in AUC (0.79), we also evaluated the precision-recovery curve. Additionally, various risk markers identified in their study using the interpretability approach coincided with the ones we found herein.

Machine learning algorithms were used to find prognostic clinical biomarkers in patients with COVID-19 in other populations at the onset of the pandemic; however, small sample sizes in those early efforts limited model robustness and performance [27–32]. Subsequent studies included larger sample sizes [33–37]. Other studies have proposed models based on laboratory tests and imaging markers; however these approaches have shown a high risk of bias, have been optimistic in their reported performance [12] and have only limited application in areas where resources are scarce [38–40].

### Sex and age as risk markers

Concurring with the findings reported by Aktar et al. [25], our results in the general population and stratified by age indicate that older age was the most significant predictor of mortality, as well. Our results confirmed that age is the strongest demographic factor for a fatal outcome in both sexes; however, the risk of COVID-19 lethality increases at a younger age in males than in females. Studies in other populations have reported age as a predictor of COVID-19 mortality [35, 41, 42]; in addition, two previous studies reported that the behavior of COVID-19 mortality and the case fatality rate with respect to age graphically resembles a J-shaped curve, which is interpreted as a minor impact of mortality at an early age and a progressive impact as age increases. This rate is higher in older populations [43, 44].

In a previous study of confirmed COVID-19 cases in Mexico, an age older than 41 years was associated with an increased risk of COVID-19 mortality, in line with our findings that the number of cases of death grew exponentially from the age group 40–49 years onwards [45]. On the other hand, Mesta et al. reported that an age older than 60 years was associated with death in patients hospitalized for COVID-19 in Mexico, which agrees with our results that lethality was concentrated in the age range of 60 years or older and in the male sex [46]. Immune senescence, characterized by progressive lymphopenia with CD4 + T cell depletion and decreased regulatory T cell function in aging, could be a factor that makes older individuals more sensitive to severe COVID-19 [33]. In recent estimates, the burden of COVID-19 in Mexico in DALYs was the highest in patients in the 60–79 age group [1].

The finding that male patients have a higher risk of adverse clinical outcomes for COVID-19 is consistent with previous reports in the literature, and may be related to a weaker immune response; indeed, mechanistic studies in human patients and animal models have shown that immune responses to respiratory virus infections are more effective in females than in males [47].

### Comorbidities as risk markers

Considering that diabetes mellitus is a major cause of morbidity in the Mexican population [48], it is crucial to determine its role as a risk marker and its contribution to COVID-19 lethality. In our study, diabetes had a greater weight in case fatality among females in most of the age groups analyzed. However, these results do not rule out the risk posed by diabetes in COVID-19 lethality for males. This could be due to the coexistence of more than one comorbidity; in these cases, hypertension and CKD are better risk markers in males and contribute more to disease lethality [49].

In 2020, Bello-Chavolla et al. developed the MSL-COVID-19 score to predict case fatality rates in patients hospitalized for COVID-19 in Mexico [24]. In that work, CKD was also reported as a comorbidity associated with COVID-19 lethality. In our study, CKD showed a significant contribution to lethality in males. However, those researchers reported that COPD and immunosuppression were risk factors significantly associated with lethal cases of COVID [38]. In the largest cohort study on the disease conducted to date by OpenSAFELY, CKD was reported to be a key risk factor for COVID-19 mortality [50, 51].However, unlike Bello-Chavolla, we stratified our population by age group and sex, and found that CKD had a higher weight in males in the 30–49 and 60–69 age groups, and in females in the 70 and older age group.

Other studies, conducted in a population of the Mexican Social Security Institute (IMSS) [52], in a population of the State of Coahuila, Mexico [53], and in individuals treated in Mexican healthcare units and hospitals [8], all of them with a sample size comparable to ours, reported hypertension, diabetes, CKD, and obesity as comorbidities that increase the risk of mortality from COVID-19 [52, 53]. Our results indicate that arterial hypertension is the comorbidity with the greatest contribution to lethality regardless of sex; interestingly, it was also a risk marker in most age-groups.

The role of diabetes and obesity as risk factors for death from COVID-19 has been consistently reported in several studies in Mexico and worldwide [48, 54–58]; However, obesity was not a prominent risk marker in our work. The combined effect of diabetes and obesity on COVID-19 lethality has been attributed to the proinflammatory state associated with both comorbidities. In addition, SARS-CoV2 infection is known to elicit a dysregulated immune response that promotes hyperinflammation and endothelial dysfunction, which may result in a prothrombotic state [59] and an excessive oxidative stress response [60].

Overall, our results are in agreement with previous studies in the Mexican population, which reported older age, male sex, hypertension, diabetes, and CKD as risk factors for death and severity in patients with COVID-19 [4, 8, 45, 46, 48, 52, 53, 61–63].

This is of utmost relevance, given that 76% of the Mexican population over 20 years of age is overweight or obese, and the prevalence of obesity increased by 42%, particularly in persons over 50 years of age [64]. Additionally, 2.8 million Mexicans have diabetes, and a prevalence of 7.6% was reported for this disease in adults aged 30–39 years (5.4,10.7), according to the National Health and Nutrition Survey (ENSANUT) 2020 [65]. Thus, the profile of metabolic risk of the Mexican population makes it extremely vulnerable to a fatal outcome from COVID-19. According to our results cardiovascular diseases were not relevant in COVID-19 lethality, same results with the study by Castelnuovo et al. where they applied machine learning and Cox regression methods in Italian population, they found no association between these diseases with COVID-19 mortality [66]. However, the study´s results by Spinoni where they applied logistic regression models and estimated the survival rate with the Kaplan-Meier method in the Italian population to specifically evaluate the association of atrial fibrillation with COVID-19 mortality, showed that atrial fibrillation increases the risk of dying compared to those who do not have it, even more so when it is an event that occurs during the course of the disease compared to those patients with a history of this cardiovascular disease, possibly due to the their population longevity and the high prevalence of 20% in these patients [67]. 

### Symptoms as risk markers

With respect to symptoms as markers of lethality, signs of respiratory distress like dyspnea and polypnea were the most frequent risk markers in all age groups. A study in Brazil reported that respiratory distress was more likely in male patients who died of COVID-19 [35]. Dyspnea, polypnea, chest pain, myalgia, arthralgia, fever, and chills were also the most frequent symptoms. In agreement with those results, dyspnea and fever were also reported by Aktar and Cini Oliveira [25, 26]. However, our results indicate that headache, odynophagia, and cough failed to contribute to lethality. Chest pain was the most important lethality marker for male patients in various age groups, whereas arthralgia was the main risk marker for females.

The interpretability approach allowed us to identify some clinical signs that decrease the risk of lethality, namely rhinorrhea and anosmia in female patients, and conjunctivitis and odynophagia in males; these signs could be related to some benign presentation of the disease. On the other hand, the finding that a more rapid attention increased the risk of lethality could be due to a cultural habit of the Mexican population to seek care immediately when the course of the disease is severe. This contrasts with the results reported by Mancilla-Galindo et al. that the risk of fatal outcome in Mexican patients with COVID-19 increased for each day of delay in receiving medical care after symptom onset, which also correlated with age [23], and by Martos et al. that longer periods elapsed from symptom onset to the start of medical care were associated with more adverse clinical outcomes, highlighting the importance of early medical consultation [62].

The use of antipyretics was a recurrent marker in males and, to a lesser extent, in females. Its risk-increasing effect on lethality could be explained by the fact that these drugs are used by patients as a default response to severe symptoms; therefore, these drugs are not a contributing variable per se, but could be indicating patient-perceived severity.

### Limitations and contributions

Our study has strengths and limitations. Its main strength is the stratification of the population and the inclusion of demographic and clinical variables accessible to clinicians in resource-limited settings. This allowed us to identify risk markers and their contribution to COVID-19 lethality, assessing their relative weight according to sex and age group. Male sex is commonly assumed to be a determining condition for disease outcome, which may lead to underestimation of risk in females.

A key contribution of this work is the development of a model for early prediction of increased lethality risk using machine learning algorithms, the use of an interpretability approach to identify markers of lethality risk by sex and age, and of the SHapley Additive exPlanations (SHAP). By analyzing predictors of COVID-19 mortality risk by sex and age group, we were able to identify those segments with the highest vulnerability. In addition, including both outpatients and inpatients allowed us to capture the full spectrum of COVID-19 cases.

Our prediction model based on sociodemographic and clinical parameters could provide a tool to select patients at higher risk of a lethal outcome from COVID-19 in a first-contact setting, especially in geographic areas where laboratory infrastructure and hospital care are limited. However, extensive external validation studies are required to assess its performance in triage prior to its clinical use.

The main limitation of our study is the quality of the information available for some variables, and the fact that some responses are not specific; for example, the variable “cardiovascular disease” encompasses several conditions, often poorly delimited; self-reporting of comorbidities could lead to an underestimation of risk, especially in subclinical patients. Data were not updated as the disease evolved, and all recorded signs and comorbidities were reported by patients when they received medical care; in addition, data on SARS-CoV-2 virus variant in confirmed cases were also missing. Finally, the database was only reviewed and validated by the Mexican Ministry of Health.

We are aware that other general variables, easily accessible to clinicians, such as comorbidity control history, medication, and vital signs, could improve the performance of our prediction model and allow us to identify more accurate risk markers. Another limitation was that our models were not validated with an external cohort. Finally, this study was conducted in a Mexican population, so caution should be taken when generalizing its results to other populations with a different demographic and metabolic profile.

## Conclusions

This study showed that age was the strongest demographic factor for a fatal outcome in both sexes. The risk of death from COVID-19 rises with age and begins to increase at an earlier age in males than in females. Age and sex set a baseline risk of death, which is higher in male patients, and this risk could increase or decrease, depending on other risk markers.

Signs of respiratory distress, such as dyspnea and polypnea, were the main predictive symptoms of COVID-19 lethality, showing a greater weight in males than in females.

Hypertension was the comorbidity with the highest contribution to lethality, irrespectively of sex. Chronic kidney disease was a major marker for males, whilst diabetes was a significant risk marker for females.

Some manifestations of the disease decreased the risk of lethality, including rhinorrhea, anosmia, conjunctivitis, and odynophagia, possibly because of their association with some benign presentation of the disease. In addition, the fact that a shorter delay in the provision of care increased the risk of lethality could be an indirect effect of the tendency of the Mexican population to seek immediate care when the course of the disease is more severe.

Herein, we demonstrate that machine learning-based models can identify risk markers for lethality by sex and age, which were consistent with the results of previous statistically based studies in the Mexican population. Finally, individual predictions could help improve our clinical understanding of COVID-19 care by providing an overview of possible outcomes for a patient based on their clinical history. Such markers have the potential to be used in triage and prognosis in the populations that lack access to highly specialized health services.

Currently, the critical stage of the COVID-19 pandemic is over. However, in order to prepare ourselves to respond to possible public health emergency of international concern (PHEIC), and considering the experience of COVID-19, it is crucial to design and implement research tools and methodologies for the next pandemic event. To our knowledge, this work is a first approach to ML-based clinical research in public health issues, and it could be valuable in a country like Mexico, where saturation of health services is the norm and where the use of ML-based approaches in public health is still incipient. We hope that this study will drive the use of machine learning approaches in future research in emergency situations, where the healthcare system requires the support of non-biomedical sciences, emphasizing the need for stratification by sex and age as a first step towards personalized care.

## Supplementary Information

**Additional file 1.** Description of clinical variables used in the experimentation.

**Additional file 2.** Lethality prediction performance.

**Additional file 3.** Risk markers by sex and age group.

## Data Availability

The open-access database used in this article is available at http://covid-19.iimas.unam.mx/. The source code from all experiments is available at https://github.com/rojas-mariano-salvador/Lethality-markers-for-COVID-19.

## Abbreviations

DALYs: Disability-adjusted life years
CFR: Case fatality rates
COPD: Chronic obstructive pulmonary disease
CKD: Chronic kidney disease
ML: Machine learning
AUC: Area under the curve, receiver operating characteristics
LR: Logistic regression
SVM: Support vector machine
RF: Random forest
XGBoost: Extreme gradient boosting
SHAP: SHapley Additive exPlanations
DT: Decision tree
GBM: Gradient boosting machine
LGBM: Light gradient boosting machine

## Footnotes

1 https://www.gob.mx/cms/uploads/attachment/file/636504/Estudio_epidemiol_gico_de_caso_sospechosos_de_enfermedad_respiratoria_viral.pdf

2 https://scikit-learn.org/stable/.

3 https://pandas.pydata.org/.

4 https://numpy.org.

5 https://jupyter.org/.
