# Supplementary material for: Lethality risk markers by sex and age-group for COVID-19 in Mexico: a cross-sectional study based on machine learning approach
Source: BMC Infect Dis. 2023 Jan 11;23:18. doi: 10.1186/s12879-022-07951-w (PMC9832420; doi:10.1186/s12879-022-07951-w)
Supplement: Supplementary file 1 — Additional file 1. Description of clinical variables used in the experimentation. [file 12879_2022_7951_MOESM1_ESM.docx]

**Appendix A. Description of clinical variables used in the experimentation**

| **Type** | **Feature** | **Description** | **Categories** |
| --- | --- | --- | --- |
| Demographic | Sex | Identifies the patient's sex. | 0 = Woman 1 = Man |
|  | Age | Identifies the patient's age in years. | Number |
| Symptom | Fever | Identifies if the patient has fever. | 0 = Without fever 1 = With fever |
|  | Cough | Identifies if the patient has cough. | 0 = Without cough 1 = With cough |
|  | Odynophagia | Identifies if the patient has odynophagia. | 0 = Without odynophagia 1 = With odynophagia |
|  | Dyspnea | Identifies if the patient has dyspnea. | 0 = Without dyspnea 1 = With dyspnea |
|  | Irritability | Identifies if the patient has irritability. | 0 = Without irritability 1 = With irritability |
|  | Diarrhea | Identifies if the patient has diarrhea. | 0 = Without diarrhea 1 = With diarrhea |
|  | Chest pain | Identifies if the patient has chest pain. | 0 = Without chest pain 1 = With chest pain |
|  | Chills | Identifies if the patient has chills. | 0 = Without chills 1 = With chills |
|  | Headache | Identifies if the patient has headache. | 0 = Without headache 1 = With headache |
|  | Myalgia | Identifies if the patient has myalgia. | 0 = Without myalgia 1 = With myalgia |
|  | Arthralgia | Identifies if the patient has arthralgia. | 0 = Without arthralgia 1 = With arthralgia |
|  | General discomfort | Identifies if the patient has general discomfort. | 0 = Without general discomfort 1 = With general discomfort |
|  | Rhinorrhea | Identifies if the patient has rhinorrhea. | 0 = Without rhinorrhea 1 = With rhinorrhea |
|  | Polypnea | Identifies if the patient has polypnea. | 0 = Without polypnea 1 = With polypnea |
|  | Vomiting | Identifies if the patient has vomiting. | 0 = Without vomiting 1 = With vomiting |
|  | Abdominal pain | Identifies if the patient has abdominal pain. | 0 = Without abdominal pain 1 = With abdominal pain |
|  | Conjunctivitis | Identifies if the patient has conjunctivitis. | 0 = Without conjunctivitis 1 = With conjunctivitis |
|  | Cyanosis | Identifies if the patient has cyanosis. | 0 = Without cyanosis 1 = With cyanosis |
|  | Sudden onset of symptoms | Identifies if the patient has sudden onset of symptoms. | 0 = Without sudden onset of symptoms 1 = With sudden onset of symptoms |
|  | Anosmia | Identifies if the patient has anosmia. | 0 = Without anosmia 1 = With anosmia |
|  | Dysgeusia | Identifies if the patient has dysgeusia. | 0 = Without dysgeusia 1 = With dysgeusia |
| Comorbidities | Diabetes | Identifies if the patient has a diagnosis of diabetes. | 0 = Without diabetes 1 = With diabetes |
|  | COPD | Identifies if the patient has a diagnosis of Chronic obstructive pulmonary disease (COPD). | 0 = Without COPD 1 = With COPD |
|  | Asthma | Identifies if the patient has a diagnosis of asthma. | 0 = Without asthma 1 = With asthma |
|  | Immunosuppression | Identifies if the patient has immunosuppression. | 0 = Without immunosuppression 1 = With immunosuppression |
|  | Hypertension | Identifies if the patient has a diagnosis of obesity. | 0 = Without obesity 1 = With obesity |
|  | Cardiovascular disease | Identifies if the patient has a diagnosis of cardiovascular diseases. | 0 = Without cardiovascular disease 1 = With cardiovascular disease |
|  | Obesity | Identifies if the patient has a diagnosis of Obesity. | 0 = Without obesity 1 = With obesity |
|  | CKD | Identifies if the patient has a diagnosis of chronic kidney disease. | 0 = Without CKD 1 = With CKD |
|  | Smoking | Identifies if the patient has a smoking habit. | 0 = Without smoking 1 = With smoking |
| Medication | Use of antipyretics | Identify if the patient uses antipyretics. | 0 = Without use of antipyretics  1 = With use of antipyretics |
| Other | Days elapsed | Is the time in days elapsed from the onset of disease symptoms to the start of medical care. | Number |
| Lethality | Mortality | Identify the outcome of the disease. This variable was the label to predict in the classification methods | 0 = Survival  1 = Mortality |
