# Supplementary material for: Lethality risk markers by sex and age-group for COVID-19 in Mexico: a cross-sectional study based on machine learning approach
Source: BMC Infect Dis. 2023 Jan 11;23:18. doi: 10.1186/s12879-022-07951-w (PMC9832420; doi:10.1186/s12879-022-07951-w)
Supplement: Supplementary file 2 — Additional file 2. Lethality prediction performance. [file 12879_2022_7951_MOESM2_ESM.docx]

***Appendix B. Lethality prediction performance***

**Figure 1.** Comparison of machine learning classification models using 10 repetitions of stratified 10-fold cross-validation.


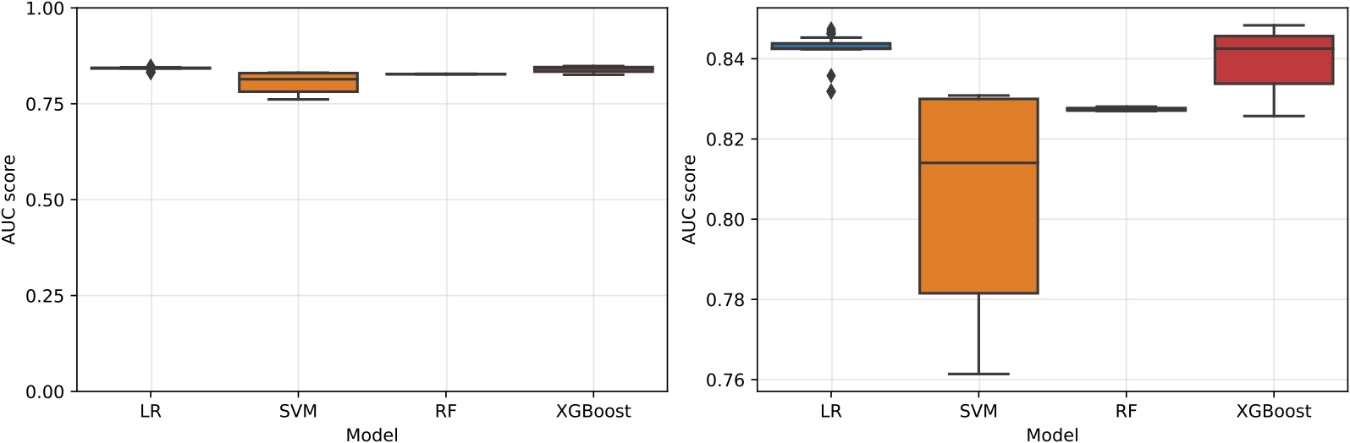


The y-axis range of the figure on the left side is from 0 to 1. In contrast, the y-axis range of the figure on the right side is from 0.76 to 0.84. Both figures represent the same information, only the scale is different.
