# Supplementary material for: Lethality risk markers by sex and age-group for COVID-19 in Mexico: a cross-sectional study based on machine learning approach
Source: BMC Infect Dis. 2023 Jan 11;23:18. doi: 10.1186/s12879-022-07951-w (PMC9832420; doi:10.1186/s12879-022-07951-w)

***Appendix C. Risk markers*** ***by sex and age group***

Figures 1-10 show the identified markers by sex and age group that increase the risk of lethality. On the one hand, the bars plots (on the left side) show the feature importance listed in order of importance, from the most important to the least important. On the other hand, the beeswarm plots (on the right side) show the impact of each feature value on the model output. In the X-axis show SHAP value: larger positive SHAP values increases the risk of lethality, and larger negative SHAP values decreases the risk. In this plot, each patient is presented as dot. Multiples dots in the same x-axis shape a density. The color indicates the value of the feature. Larger values of the features is denoted as red color, and lower values is indicated as blue color.

**Figure 1.** SHAP approach for males age group 30–39 years.


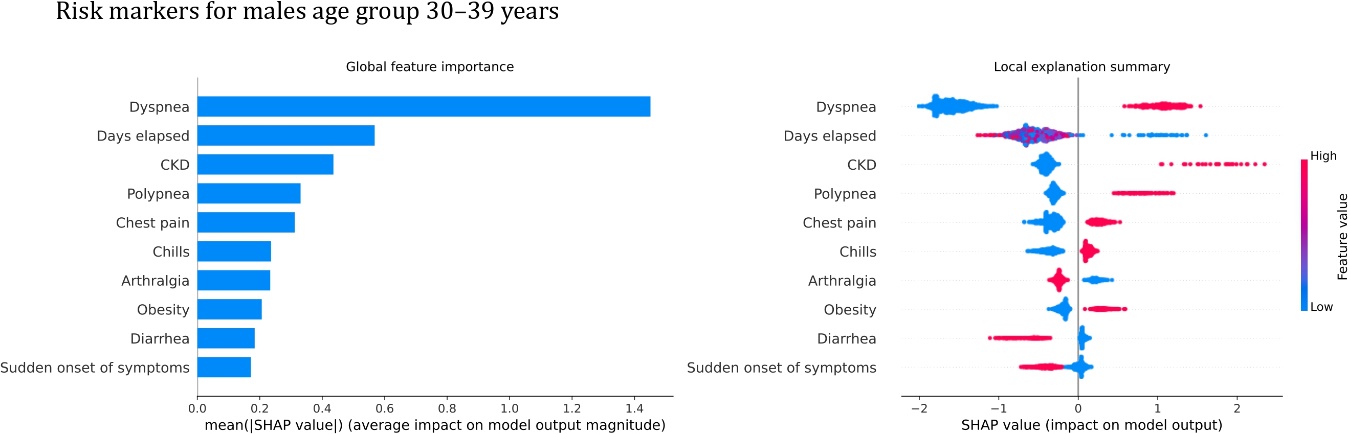


**Figure 2.** SHAP approach for males age group 40–49 years.


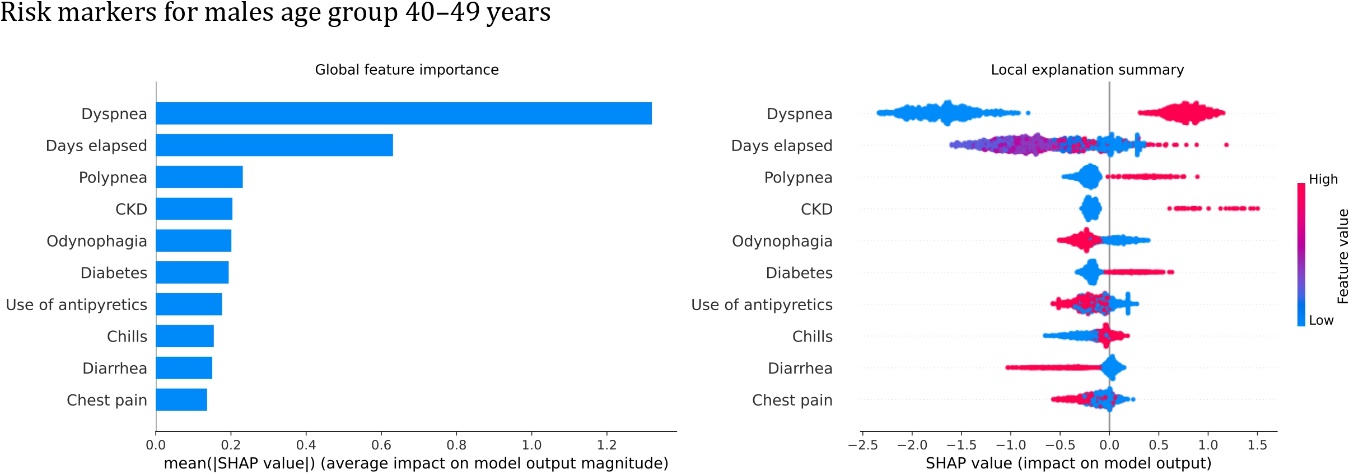


**Figure 3.** SHAP approach for males age group 50–59 years.


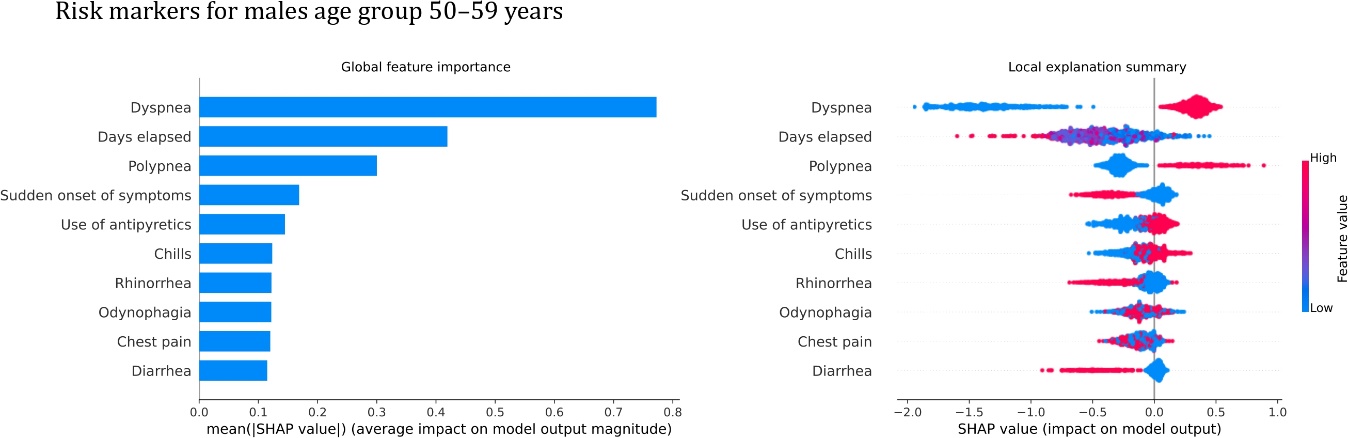


**Figure 4.** SHAP approach for males age group 60–69 years.


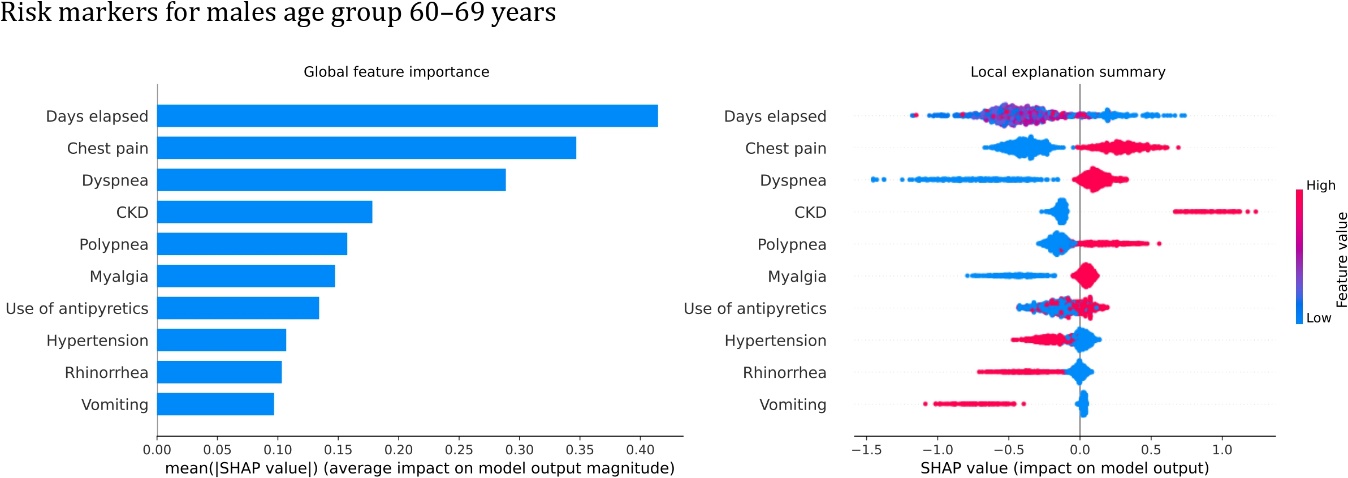


**Figure 5.** SHAP approach for males age group 70 years and over.

**
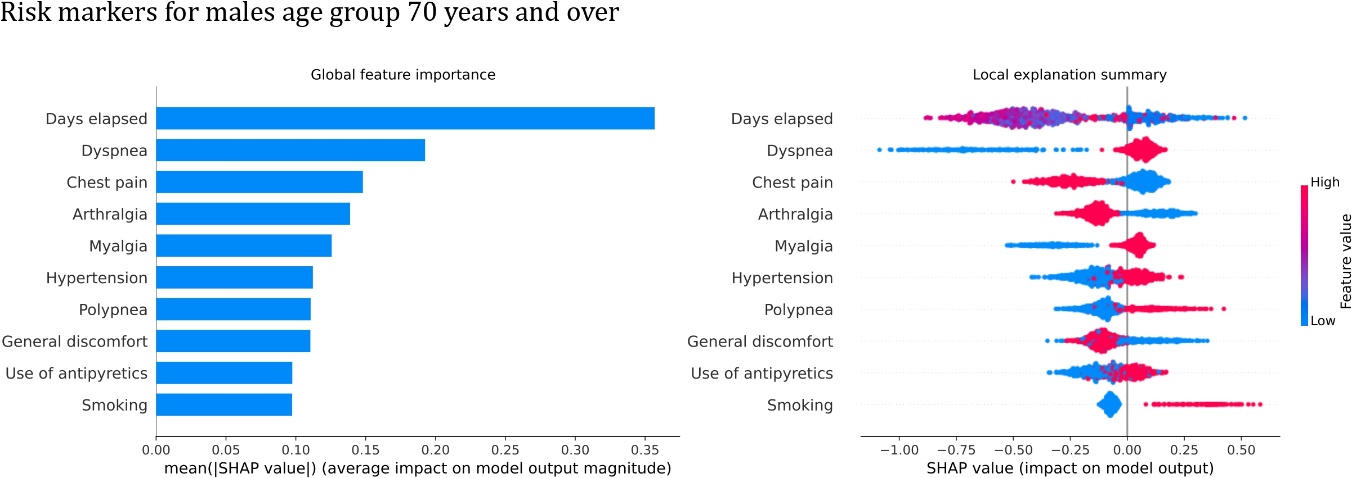
**

**Figure 6.** SHAP approach for females age group 30–39 years.

**
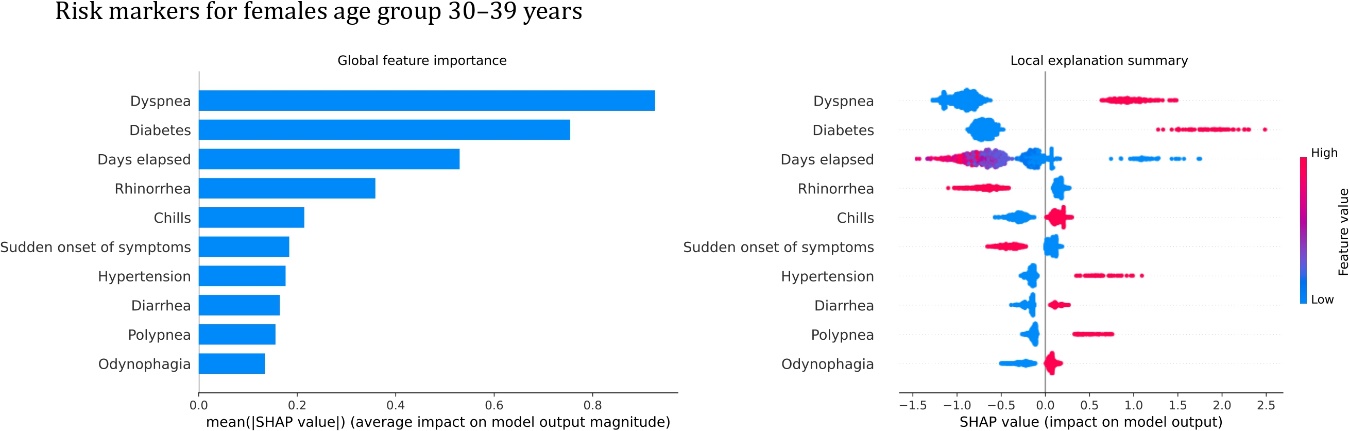
**

**Figure 7.** SHAP approach for females age group 40–49 years.

**
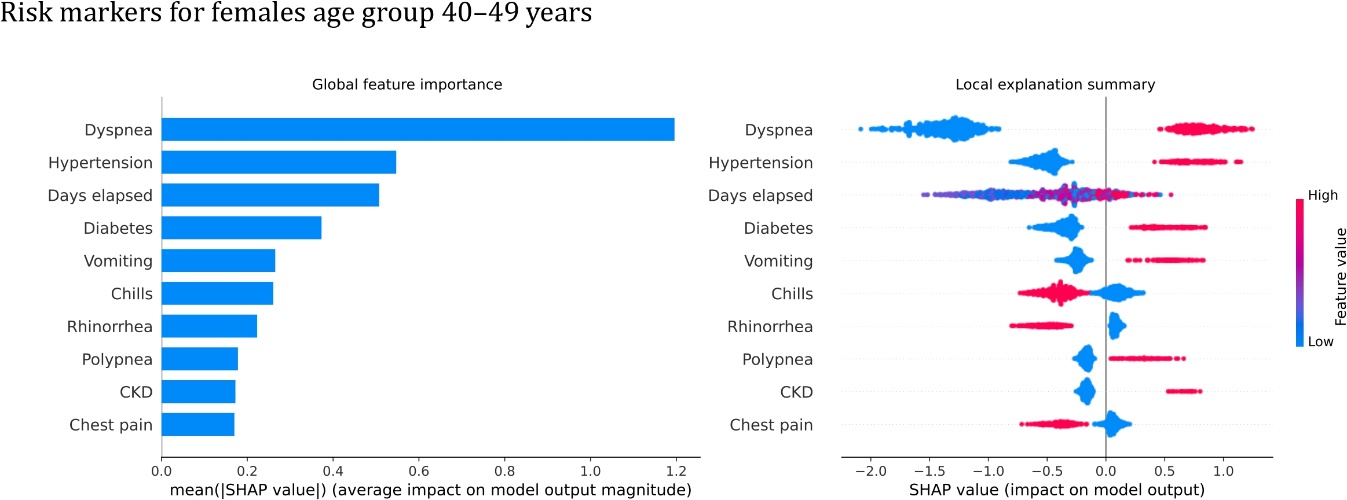
**

**Figure 8.** SHAP approach for females age group 50–59 years.


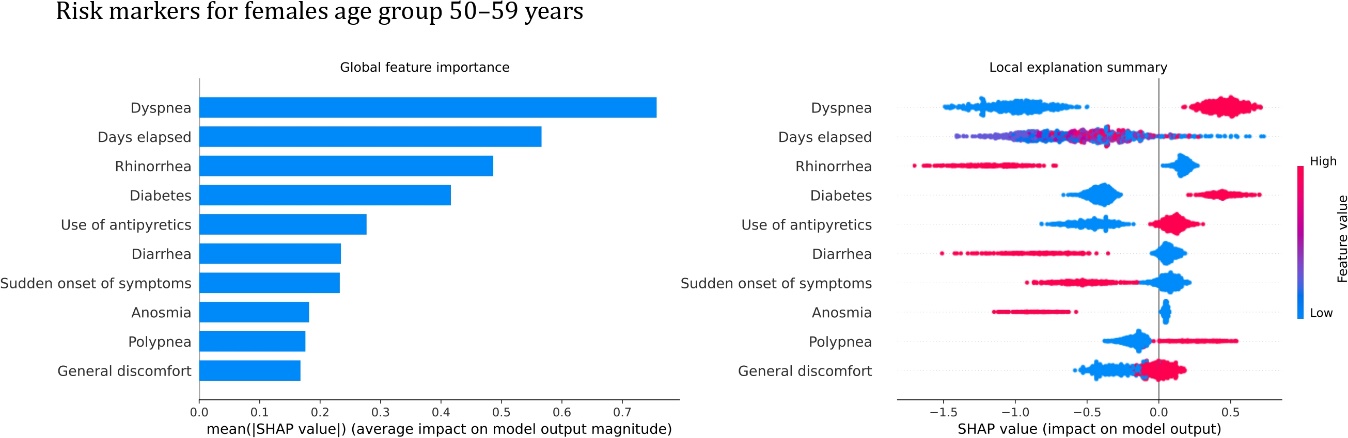


**Figure 9.** SHAP approach for females age group 60–69 years.

**
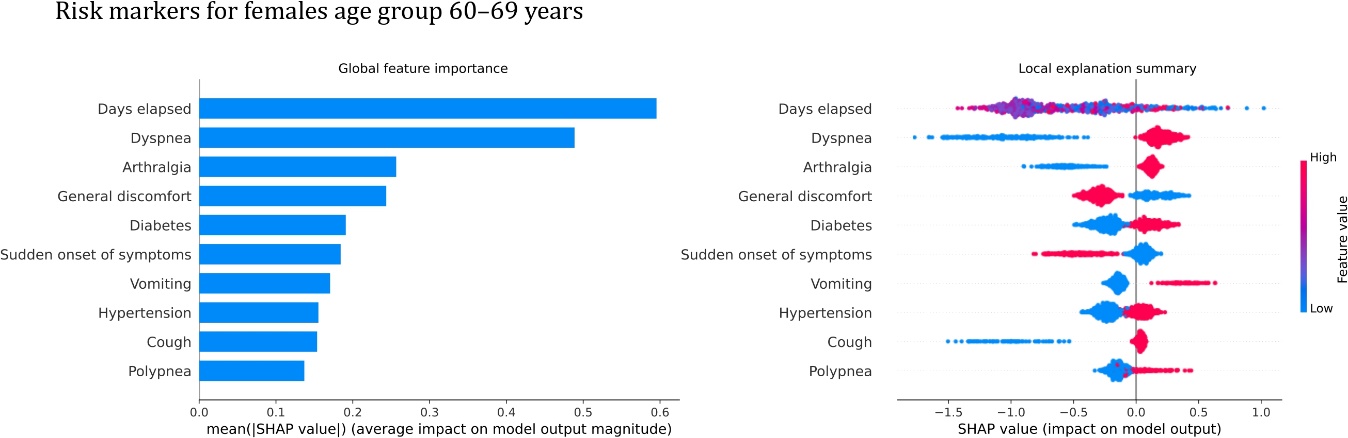
**

**Figure 10.** SHAP approach for females age group 70 years and over.


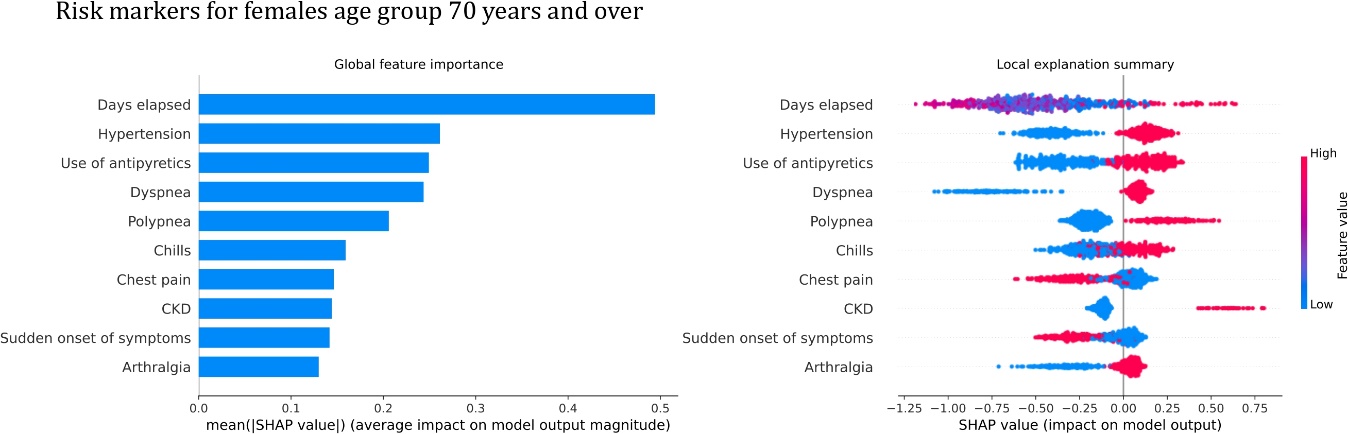

Supplement: Supplementary file 3 — Additional file 3. Risk markers by sex and age group. [file 12879_2022_7951_MOESM3_ESM.docx]
